# Bioinformatic analyses hinted at augmented T helper 17 cell differentiation and cytokine response as the central mechanism of COVID‐19–associated Guillain‐Barré syndrome

**DOI:** 10.1111/cpr.13024

**Published:** 2021-03-10

**Authors:** Zheng Li, Ziheng Huang, Xingye Li, Cheng Huang, Jianxiong Shen, Shugang Li, Lin Zhang, Sunny H. Wong, Matthew T. V. Chan, William Ka Kei Wu

**Affiliations:** ^1^ Department of Orthopaedic Surgery Peking Union Medical College Hospital Chinese Academy of Medical Sciences and Peking Union Medical College Beijing China; ^2^ CUHK‐Shenzhen Research Institute Shenzhen China; ^3^ Department of Orthopedic Surgery Beijing Jishuitan Hospital Fourth Clinical College of Peking University Jishuitan Orthopaedic College of Tsinghua University Beijing China; ^4^ Department of Orthopaedic Surgery Center for Osteonecrosis and Joint Preserving & Reconstruction China‐Japan Friendship Hospital Beijing China; ^5^ Department of Anaesthesia and Intensive Care Peter Hung Pain Research Institute The Chinese University of Hong Kong Hong Kong China; ^6^ State Key Laboratory of Digestive Disease LKS Institute of Health Sciences The Chinese University of Hong Kong Hong Kong China; ^7^ Department of Medicine and Therapeutics The Chinese University of Hong Kong Hong Kong China

**Keywords:** COVID‐19, Guillain‐Barré syndrome, macrophage, SARS‐CoV‐2

## Abstract

**Objectives:**

Guillain‐Barré syndrome (GBS) results from autoimmune attack on the peripheral nerves, causing sensory, motor and autonomic abnormalities. Emerging evidence suggests that there might be an association between COVID‐19 and GBS. Nevertheless, the underlying pathophysiological mechanism remains unclear.

**Materials and Methods:**

We performed bioinformatic analyses to delineate the potential genetic crosstalk between COVID‐19 and GBS.

**Results:**

COVID‐19 and GBS were associated with a similar subset of immune/inflammation regulatory genes, including *TNF*, *CSF2*, *IL2RA*, *IL1B*, *IL4*, *IL6* and *IL10*. Protein‐protein interaction network analysis revealed that the combined gene set showed an increased connectivity as compared to COVID‐19 or GBS alone, particularly the potentiated interactions with *CD86*, *IL23A*, *IL27*, *ISG20*, *PTGS2*, *HLA‐DRB1*, *HLA‐DQB1* and *ITGAM*, and these genes are related to Th17 cell differentiation. Transcriptome analysis of peripheral blood mononuclear cells from patients with COVID‐19 and GBS further demonstrated the activation of interleukin‐17 signalling in both conditions.

**Conclusions:**

Augmented Th17 cell differentiation and cytokine response was identified in both COVID‐19 and GBS. PBMC transcriptome analysis also suggested the pivotal involvement of Th17 signalling pathway. In conclusion, our data suggested aberrant Th17 cell differentiation as a possible mechanism by which COVID‐19 can increase the risk of GBS.

## Graphical abstract

Functional protein‐protein association networks of (A) COVID‐19– and (B) GBS‐related genes were analyzed by STRING. Genes associated with the two disease entities (COVID‐19, disease id: C000657245, version 4, N.PMIDs ≥ 4; GBS, disease id: C0018378, Gene‐Disease Association Score ≥ 0.02) were retrieved from DisGeNET, a knowledge platform for disease genomics. Only highly confident interactions derived from ‘Experiments’, ‘Databases’, ‘Co‐expression’ and ‘Co‐occurrence’ with the minimum required interaction score of 0.900 were shown. The Markov cluster (MLC) algorithm with an inflation parameter of 3 was used for network clustering.

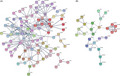

## 1 INTRODUCTION

As of 13 January 2021, over 91 million patients have been diagnosed of coronavirus disease 2019 (COVID‐19), causing more than 1.96 million deaths worldwide. The causative agent, severe acute respiratory syndrome coronavirus 2 (SARS‐CoV‐2), was found to mediate host cell entry by binding to the host receptors ACE2 and then propagate intracellularly, causing the symptoms of COVID‐19.^1^, ^2^, ^3^ Male gender, advanced age, cigarette smoking and the presence of comorbidities (eg hypertension, diabetes) have been associated with a heightened risk of unfavourable outcomes.^4^, ^5^ Although the exact molecular pathogenesis of COVID‐19 is still being investigated, deranged immune response rather than SARS‐CoV‐2 itself seems to cause the worst damage in some severe cases.^6^ Aside from respiratory symptoms, increasing amount of evidence suggested that COVID‐19 might influence both the peripheral and central nervous systems, increasing the risks of ischaemic stroke, meningoencephalitis, acute necrotizing encephalopathy, spinal cord dysfunction and Guillain‐Barré syndrome (GBS).^7^, ^8^ Radiological manifestations of COVID‐19–associated neurological defects include nerve root enhancement, microhaemorrhages or infarcts.

Guillain‐Barré syndrome encompasses a spectrum of autoimmune diseases of the peripheral nervous system, namely acute inflammatory demyelinating polyradiculoneuropathy (AIDP), acute motor axonal neuropathy (AMAN), acute motor‐sensory axonal neuropathy (AMSAN), Miller Fisher syndrome and acute pandysautonomic neuropathy. AIDP is the most common clinical subtype of GBS (85% of cases) and is characterized pathologically by T cell–mediated activation of macrophages that invade the myelin sheaths, causing denudation of the axons (ie demyelination).^9^, ^10^ In contrast, B cell–mediated humoral immunity is strongly associated with AMAN and Miller Fisher syndrome, in which autoantibodies are produced to bind to gangliosides GM1 and GD1a at the nodes of Ranvier and/or anti‐GQ1b in the paranodal region.^11^, ^12^ Depending on the type of peripheral nerve involved, patients of GBS could present with paraesthesia or pain, acute symmetric ascending motor weakness (the most severe manifestation is respiratory failure due to respiratory muscle weakness) or autonomic abnormalities (eg orthostatic hypotension, disturbances of heart rate and rhythm).^13^, ^14^ Infections with *Campylobacter jejuni* and cytomegalovirus are two major risk factors of GBS.^15^, ^16^ In *C* *jejuni*–associated GBS cases, molecular mimicry between the ganglioside‐like structures in lipo‐oligosaccharides of the bacterium and gangliosides on the axonal surface contributes to the cross‐reactivity of autoantibodies.^17^, ^18^ Nevertheless, it is noteworthy that not all *C* *jejuni*–infected individuals develop GBS, suggesting that other mechanisms are at work.^19^ In this regard, derangement of other components of the immune system has been suggested to play a pivotal role in the molecular pathogenesis of GBS. For instance, circulating T helper (Th) 17, Th22, and Th1 cells were elevated,^20^ whereas regulatory T cells (Tregs) were reduced in patients with GBS.^21^ Polymorphisms in genes encoding cytokines, such as tumour necrosis factor‐α and interleukin (IL)‐10, were also associated with susceptibility to GBS.^22^

Emerging studies have indicated that there is an epidemiological linkage between COVID‐19 and autoimmune/autoinflammatory diseases, such as paediatric inflammatory multisystemic syndrome.^23^ In particular, a recent systematic review supported that GBS is emerging as a disease that may appear in COVID‐19–infected patients^23^ that has a strong autoimmune/autoinflammatory component. Electromyographic findings of axonal injury and demyelination have been observed in some COVID‐19–associated patients with GBS.^10^, ^24^, ^25^ As the pandemic of COVID‐19 continues, clinical features of COVID‐19–associated GBS will continue to be clarified. However, the exact pathophysiological mechanisms underlying this disease entity remain unknown. In this study, we performed bioinformatic analyses to delineate the potential crosstalk between COVID‐19 and GBS‐associated genes, which can illuminate the pathogenic mechanisms of COVID‐19–associated GBS to derive potential therapeutics for its treatment.

## 2 MATERIALS AND METHODS

### 2.1 Identification of COVID‐19– and GBS‐associated genes

Information of genes associated with these two disease entities (COVID‐19, disease id: C000657245, version 4, N.PMIDs ≥ 4; GBS, disease id: C0018378, Gene‐Disease Association Score ≥ 0.02) was retrieved from DisGeNET, a knowledge platform for disease genomics. These gene sets were subject to functional protein association networks analysis using STRING to look for missing genes in the networks to produce the final gene sets.

### 2.2 Protein‐protein interaction networks of COVID‐19– and GBS‐associated genes

Protein‐protein interaction network analysis using STRING was visualized with the minimum required interaction score of 0.900 (the highest confidence) and the disconnected nodes hidden in the network. Only ‘Experiments’, ‘Databases’, ‘Co‐expression’ and ‘Co‐occurrence’ were chosen as the active interaction sources. The Markov cluster (MLC) algorithm, which is a scalable unsupervised cluster algorithm based on simulation of stochastic flow in networks, with an inflation parameter of 3 was used for network clustering.

### 2.3 Transcriptome analysis

RNA sequencing data sets of peripheral blood mononuclear cells (PMBCs) isolated from 3 patients with COVID‐19 and 3 healthy controls were obtained from Genome Sequence Archive in BIG Data Center (https://bigd.big.ac.cn/) under the project PRJCA002326. RNA sequencing data sets of PBMCs isolated from a patient with GBS and her healthy twin at time point 2 during intermediate care were obtained from the Gene Expression Omnibus (GEO) within the series GSE72748 (http://www.ncbi.nlm.nih.gov/geo/query/acc.cgi?acc=GSE72748). RNA sequencing data were first evaluated by FastQC (https://www.bioinformatics.babraham.ac.uk/projects/fastqc/) with the default parameter and then aligned with Hisat2 (version 2.1.0) onto the human (hg38) genome guided by GENCODE gene annotation (version 34) with the default parameter. The expression of genes in each sample was calculated by the featureCounts packages (version 2.0.1) with the ‘‐M’ parameter. Differentially expressed genes were identified using the R package EBSeq (version 1.28.0) with the following conditions: adjusted *P*‐value < .05 and the absolute value of log_2_ fold change >1.

### 2.4 Function and pathway enrichment analyses

Kyoto Encyclopedia of Genes Genomes (KEGG) enrichment analysis and the false discovery rate (FDR) calculation were performed with the built‐in function of STRING to infer the potential biological functions of COVID‐19– and GBS‐associated gene networks.

## 3 RESULTS

### 3.1 Protein‐protein interaction networks of both COVID‐19– and GBS‐associated genes showed enrichment in immune/inflammation‐related pathways

**FIGURE 1:**
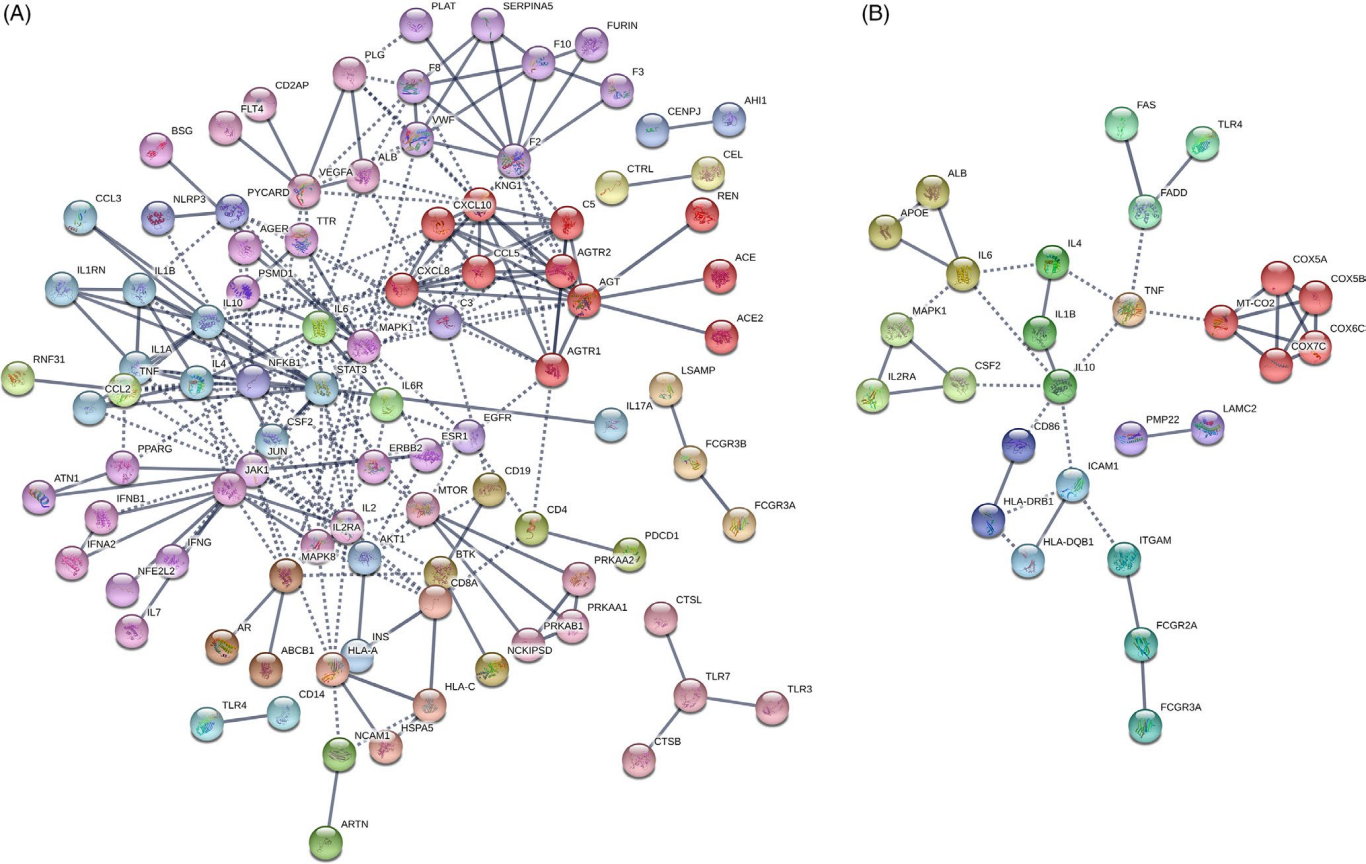
Functional protein‐protein association networks of (A) COVID‐19 and (B) GBS‐related genes were analysed by STRING. Genes associated with the two disease entities (COVID‐19, disease id: C000657245, version 4, N.PMIDs ≥ 4; GBS, disease id: C0018378, Gene‐Disease Association Score ≥ 0.02) were retrieved from DisGeNET, a knowledge platform for disease genomics. Only highly confident interactions derived from ‘Experiments’, ‘Databases’, ‘Co‐expression’ and ‘Co‐occurrence’ with the minimum required interaction score of 0.900 were shown. The Markov cluster (MLC) algorithm with an inflation parameter of 3 was used for network clustering

We first defined the list of genes involved in the pathogenesis of COVID‐19 and GBS using a disease‐based pathway analysis. High‐confident genes associated with COVID‐19 (disease id: C000657245, version 4; N.PMIDs ≥ 4) and GBS (disease id: C0018378; Gene‐Disease Association Score ≥ 0.02), respectively, were identified with DisGeNET. We then reconstructed the functional protein‐protein association networks among these genes using STRING and identified additional connecting genes (Table 1; Figure 1A for COVID‐19; Figure 1B for GBS). IL‐10 assumed a high connectivity in both COVID‐19– and GBS‐associated networks with number of interacting partners of 13 and 6, respectively. Functional prediction further indicated that immune/inflammation‐associated pathways, including ‘IL‐17 signalling pathway’, ‘Th17 cell differentiation’, ‘cytokine‐mediated receptor interaction’ and ‘TNF signalling pathway’, were significantly enriched in both gene networks (all FDR < 0.0001; Table 2).

**TABLE 1:** Disease‐associated genes retrieved from DisGeNET

Diseases	Disease‐associated genes
COVID‐19	*CD4*, *KRT20*, ***MAPK1***, *STS*, *PLAT*, *AMH*, *CRX*, *C5*, *CCL2*, *NFKB1*, *IL2*, *IFNG*, *PRKAB1*, ***IL4***, *TTR*, *C3*, *PYCARD*, *MAS1*, ***CRP***, *IL1RN*, *VWF*, *SPECC1*, *FLT4*, *SLC27A5*, *IL1A*, ***IL1B***, *IL7*, *STAT3*, *KNG1*, *LAMP3*, *RTN1*, *ERBB2*, *REN*, *EGFR*, *IFNA1*, *NAAA*, *ZFYVE9*, *PPARG*, *ACE*, *AGRP*, *NCKIPSD*, ***ALB***, *TLR3*, ***CSF2***, *CD14*, *CXCL10*, *CXCL8*, *F2*, *PLG*, *PSMD1*, *RNF31*, *HBA1*, *HSPA5*, *PKD2L1*, *KLK4*, *SLC5A2*, *CALCA*, *SERPINA5*, *BSG*, *F3*, *PDCD1*, *NLRP3*, *RTN4*, *TNNI3*, *JAK1*, ***IL17A***, *CTSL*, *CTSB*, *PRKAA1*, *ATN1*, *CD2AP*, *F8*, *PARP9*, *DPP4*, *MTOR*, *MOK*, *AGT*, *SOAT1*, *AHI1*, ***FCGR3A***, *IL6R*, *GOT1*, *JUN*, *PRKAA2*, *AGTR2*, *CEL*, *SCAI*, ***TLR4***, *AR*, *AGER*, *F10*, *HLA‐C*, *CDSN*, *NPPB*, *RPGR*, ***IL2RA***, *IFNA2*, *IFNB1*, *TLR7*, *CENPJ*, *G6PD*, *GPT*, *BRD2*, *MAPK8*, *HLA‐A*, *NFE2L2*, *INS*, *MB*, *TMPRSS2*, *GGT1*, ***IL6***, *GGT2*, *CD8A*, *ARTN*, *ACE2*, ***TNF***, *ESR1*, ***IL10***, *LSAMP*, *AGTR1*, *MS4A1*, *FCGR3B*, *CD19*, *AKT1*, *CTRL*, *NELFCD*, *CCL5*, *GGTLC3*, *CCL3*, *ABCB1*, *IGHV4‐38‐2*, *VEGFA*, *CD177*, *NCAM1*, *ABO*, *FURIN*, *BTK*, *CD48*
GBS	***MAPK1*** , *IL23A*, ***IL4***, *APOE*, ***CRP***, *C19orf10*, ***IL1B***, *LAMC2*, *ICAM1*, *FCGR2A*, *CD1A*, *AIRE*, ***ALB***, ***CSF2***, *IL17D*, *ISG20*, *CD86*, ***IL17A***, *NFASC*, *FAS*, *IL27*, *HLA‐DRB1*, *MT‐CO2*, *PTGS2*, ***FCGR3A***, *CD1E*, *CD1B*, *CD1C*, ***TLR4***, *HLA‐DQB1*, ***IL2RA***, ***IL6***, ***TNF***, ***IL10***, *ITGAM*, *GFAP*, *PMP22*

Note

Genes associated with both COVID‐19 and GBS are bolded. COVID‐19, coronavirus disease 2019; GBS, Guillain‐Barré syndrome.

**TABLE 2:** Significantly enriched ‘KEGG pathways’ in COVID‐19– and GBS‐associated gene networks

Diseases	KEGG pathways of disease‐associated gene networks
COVID‐19	**Toll‐like receptor signalling pathway**, **cytokine‐cytokine receptor interaction**, PI3K‐Akt signalling pathway, **NOD‐like receptor signalling pathway**, **Th17 cell differentiation**, **haematopoietic cell lineage**, **Jak‐STAT signalling pathway**, **IL‐17 signalling pathway**, **osteoclast differentiation**, HIF‐1 signalling pathway, necroptosis, **T‐cell receptor signalling pathway**, **natural killer cell–mediated cytotoxicity**, complement and coagulation cascades, insulin resistance, **TNF signalling pathway**, adipocytokine signalling pathway, EGFR tyrosine kinase inhibitor resistance, renin‐angiotensin system, cytosolic DNA‐sensing pathway, **Th1 and Th2 cell differentiation**, antigen processing and presentation, FoxO signalling pathway, MAPK signalling pathway, **Fc epsilon RI signalling pathway**, RIG‐I–like receptor signalling pathway, longevity regulating pathway, autophagy—animal, **NF‐kappa B signalling pathway**, prolactin signalling pathway, B‐cell receptor signalling pathway, **phagosome**, cellular senescence, ErbB signalling pathway, apelin signalling pathway, insulin signalling pathway, longevity regulating pathway—multiple species, apoptosis, chemokine signalling pathway, phospholipase D signalling pathway, focal adhesion, **intestinal immune network for IgA production**, AMPK signalling pathway
GBS	**Haematopoietic cell lineage**, **IL‐17 signalling pathway**, **Th17 cell differentiation**, **cytokine‐cytokine receptor interaction**, **TNF signalling pathway**, **Jak‐STAT signalling pathway**, **intestinal immune network for IgA production**, **Toll‐like receptor signalling pathway**, **natural killer cell–mediated cytotoxicity**, **NF‐kappa B signalling pathway**, **T‐cell receptor signalling pathway**, **osteoclast differentiation**, cell adhesion molecules (CAMs), **phagosome**, **Fc epsilon RI signalling pathway**, **NOD‐like receptor signalling pathway**, **Th1 and Th2 cell differentiation**

Note

KEGG pathways are ranked according to the false discovery rate (all <0.0001) in an ascending order. Pathways under the category of ‘Human Diseases’ were omitted. Pathways associated with both COVID‐19 and GBS are bolded. COVID‐19, coronavirus disease 2019; GBS, Guillain‐Barré syndrome.

### 3.2 Additive and potentiating interactions between COVID‐19– and GBS‐associated gene networks

**FIGURE 2:**
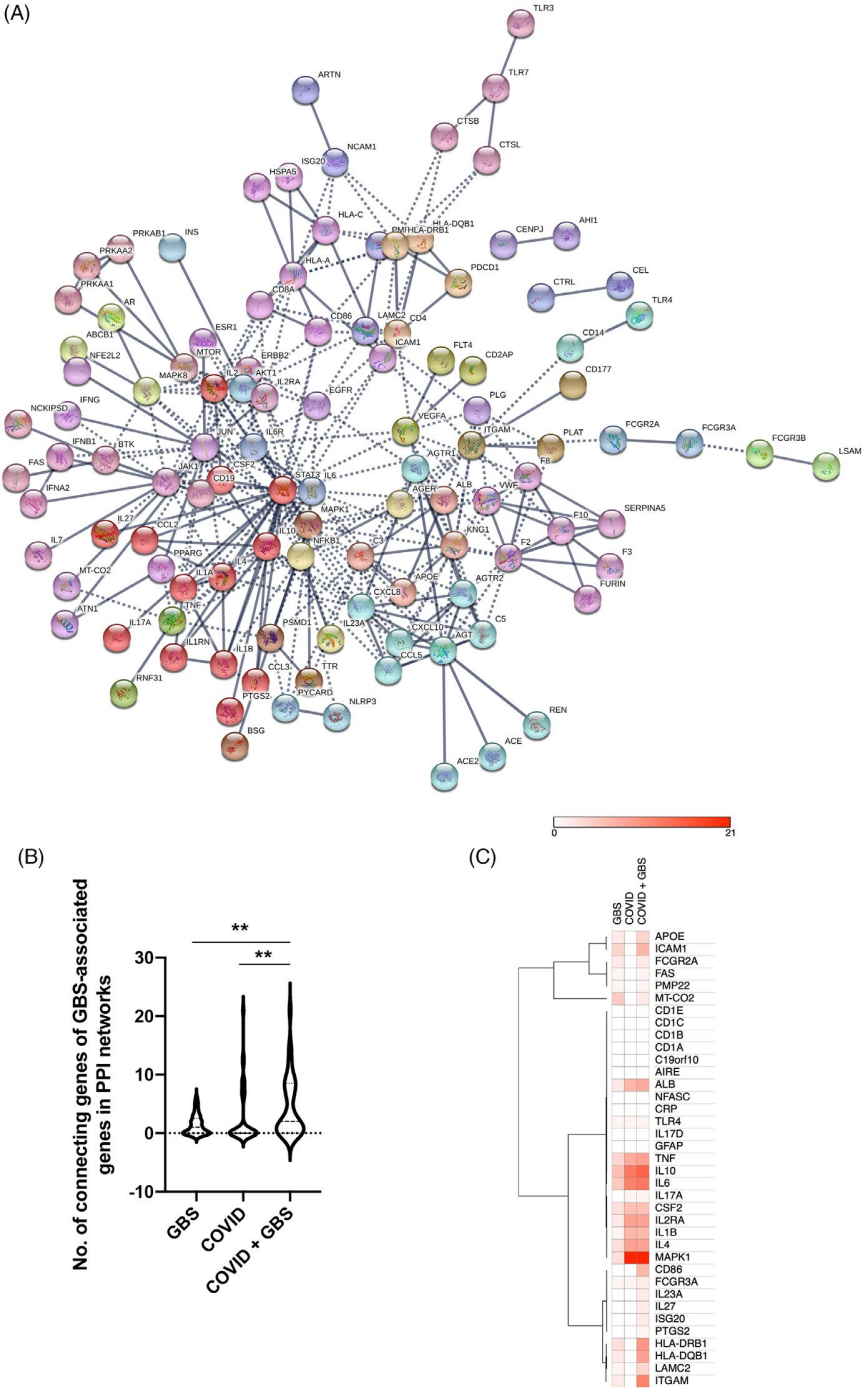
STRING analysis of combined COVID‐19/GBS gene sets. A, Interaction map shows better integration of COVID‐19– and GBS‐associated clusters. B, GBS‐associated genes showed higher connectivity in the combined network as compared to two disease entities alone. C, Heap map visualizes the additive and potentiating effects of COVID‐19–associated genes on the network connectivity of GBS‐associated genes. **, *P* < .01, significantly different between the indicated groups by non‐parametric paired analysis of the number of connecting genes of GBS‐associated genes in the protein‐protein interaction networks using Wilcoxon matched‐pairs signed rank test

To discern the potential interactions between the COVID‐19– and GBS‐associated gene networks, two gene sets were combined and subject to STRING analysis. The combined gene network showed an obvious better integration than that of COVID‐19– and GBS‐associated genes alone, with several clusters previously detached from the main network (eg TLR7‐CTSL/CTSB/TLR3, LSAMP‐FCGR3B‐FCGR3A, TLR4‐CD14 clusters in COVID‐19; PMP22‐LAMC2 cluster in GSB) now rejoined (Figure 2A). Notably, the GBS‐COVID‐19–combined networks had the highest average node degree (the number of edges connected to the node) as compared to GBS‐alone and COVID‐19‐alone networks, suggesting a synergistic interaction. To quantitatively assess the impact of COVID‐19 on GBS‐associated gene networks, number of genes connecting with the 37 GBS‐associated genes was determined in GBS‐alone, COVID‐19–alone and GBS‐COVID‐19–combined networks. The 37 GBS‐associated genes were connected to significantly more genes in the combined network as compared to two disease entities alone (Figure 2B). Visualization and clustering of such connectivity with heap map indicated two distinct patterns of interactions, namely additive (eg *ALB*, *TNF*, *CSF2*, *IL2RA*, *IL1B*, *IL4*, *IL6* and *IL10*) and potentiating (ie *CD86*, *IL23A*, *IL27*, *ISG20*, *PTGS2*, *HLA‐DRB1*, *HLA‐DQB1*, LAMC2 and *ITGAM*) interactions (Figure 2C). Gene set enrichment analysis of these 8 GBS‐associated genes with potentiating interactions with COVID‐19 implicated aberrant Th17 cell differentiation (FDR = 0.0032) was involved in COVID‐19–associated GBS.

### 3.3 Protein‐protein interaction networks of differentially expressed genes in PMBCs isolated from COVID‐19 and GBS patients

**FIGURE 3:**
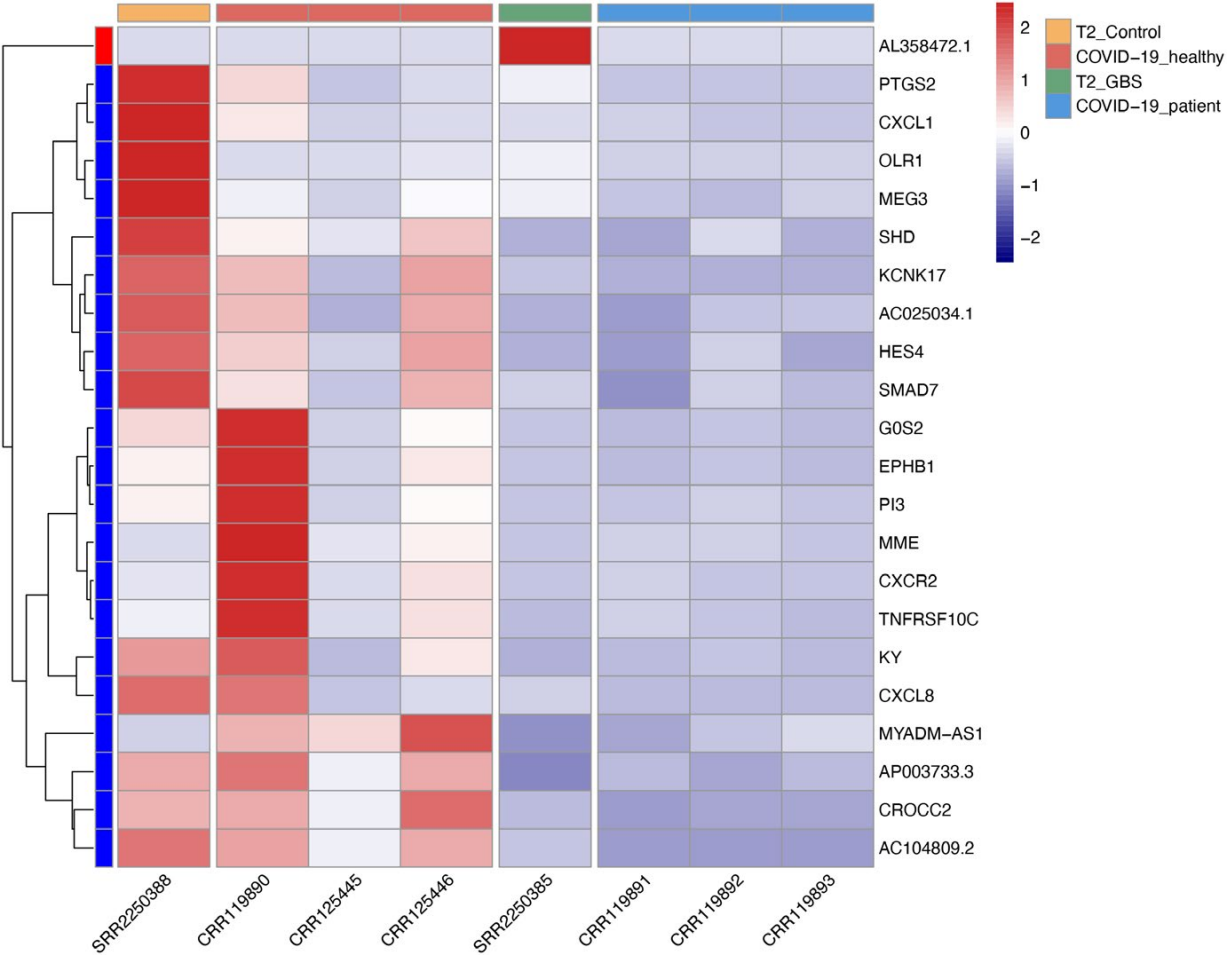
Heatmap of overlapped differentially expressed genes with concordant deregulation in PMBCs isolated from patients with COVID‐19 and GBS

To consolidate our findings, we downloaded publicly available RNA sequencing data sets of PMBCs isolated from patients with COVID‐19 and GBS and performed additional analysis. In patients with COVID‐19, a total of 1,928 differentially expressed genes (869 upregulated and 1059 downregulated) with adjusted *P*‐value < .05 and the absolute value of log_2_ fold change >1 were identified. These COVID‐19–associated differentially expressed genes were significantly enriched in pathways related to immune regulation, including ‘cytokine‐cytokine receptor interaction’, ‘Th1 and Th2 cell differentiation’ and ‘Th17 cell differentiation’ (Table 3). In GBS, a total of 447 differentially expressed genes (58 upregulated and 389 downregulated) in PBMCs were identified, which were also significantly enriched in immune‐related pathways, including ‘cytokine‐cytokine receptor interaction’, ‘IL‐17 signalling pathway’, ‘TNF signalling pathway’ and ‘Th17 cell differentiation’ (Table 3). Overlap of two gene sets identified 22 differentially expressed genes (1 upregulated and 21 downregulated) with concordant deregulation in both COVID‐19 and GBS (Figure 3A). These overlapped genes were also significantly enriched in ‘cytokine‐cytokine receptor interaction’ and ‘IL‐17 signalling pathway’ (both FDR < 0.005). These findings are consistent with aberrant Th17 cell differentiation in COVID‐19–associated GBS.

**TABLE 3:** Significantly enriched ‘KEGG pathways’ with differentially expressed genes in COVID‐19 and GBS peripheral blood mononuclear cells (PBMCs)

Diseases	KEGG pathways of disease‐associated differentially expressed genes in PBMCs
COVID‐19	**Cytokine‐cytokine receptor interaction**, carbon metabolism, cell cycle, lysosome, glycolysis / gluconeogenesis, metabolic pathways, HIF‐1 signalling pathway, amino sugar and nucleotide sugar metabolism, Th1 and Th2 cell differentiation, **Th17 cell differentiation**
GBS	**Cytokine‐cytokine receptor interaction**, IL‐17 signalling pathway, complement and coagulation cascades, TNF signalling pathway, haematopoietic cell lineage, osteoclast differentiation, NOD‐like receptor signalling pathway, NF‐kappa B signalling pathway, focal adhesion, chemokine signalling pathway, MAPK signalling pathway, TGF‐beta signalling pathway, PI3K‐Akt signalling pathway, p53 signalling pathway, Toll‐like receptor signalling pathway, **Th17 cell differentiation**

Note

KEGG pathways are ranked according to the false discovery rate (all <0.05) in an ascending order. Pathways under the category of ‘Human Diseases’ were omitted. Pathways associated with both COVID‐19 and GBS are bolded. COVID‐19, coronavirus disease 2019; GBS, Guillain‐Barré syndrome.

## 4 DISCUSSION

SARS‐CoV‐2 was found to bind to ACE2 receptor through subunit of N‐terminal S1, which was cleaved with TMPRSS2 to expose C‐terminal S2 subunit that causes cell‐virus fusion.^26^, ^27^ SARS‐CoV‐2 may impair the nervous system through 2 potential ways: maladaptive inflammatory/immune responses and direct invasion into the neural tissue. SARS‐CoV‐2 may influence nervous system via neuronal retrograde route and the haematogenous route. In this connection, ACE2 was also shown to be enriched in endothelium cells of the nervous system.^28^ Herein, we inferred the pathogenetic interactions between GBS and COVID‐19 via an integrated bioinformatic approach, which may shed novel light on the involvement of immune dysregulation, particularly aberrant Th17 cell differentiation in COVID‐19–associated GBS. However, ACE2 did not come up in our disease‐associated gene or differentially expressed gene analyses, suggesting that ACE2 might not assume a central role in the pathogenesis of COVID‐19–associated GBS. This highlights the important role of dysfunctional inflammatory/immune response, rather than direct neurological invasion in its disease mechanisms.

Guillain‐Barré syndrome is a spectrum of diseases affecting peripheral nervous system, which commonly involve axonal and demyelinating neuropathy. AIDP is the most prevalent type of GBS. One hallmark of AIDP pathogenesis is a significantly elevated cerebrospinal fluid protein level and is characterized by activated T‐cell and antibody responses to myelin epitopes in peripheral nerves. Although the exact pathophysiology of COVID‐19–associated GBS is less understood, recent studies have shown similar clinical patterns and electrophysiological subtypes with that of classic GBS. Different subtypes have been observed, although there was a preponderance for the demyelinating polyneuropathy subtype.^10^, ^25^, ^29^ Furthermore, the interval between the onset of COVID‐19 and GBS symptoms was similar to that of other established infections, such as *C jejuni* and cytomegalovirus.^24^ These observations support our approach of studying classic GBS, as a surrogate to understand the pathogenesis of COVID‐19–associated GBS, which occurs in a significant subset of patients.

Th17 cells, characterized by production of IL‐17, are a major T‐cell subset identified in 2005. Th17 plays a crucial role in production of inflammatory cytokines and recruitment of neutrophils and therefore is implicated in many autoimmune and inflammatory diseases.^30^ In patients with GBS, aside from elevated number of circulating Th17 cells, plasma IL‐17A and IL‐22 levels were increased.^31^, ^32^ Three key Th17 cytokines (IL‐6, IL‐17A and IL‐22) were also found to be elevated in the cerebrospinal fluid.^33^ Experimental autoimmune neuritis (EAN) is an animal model that recapitulates clinical, electrophysiological and immunological aspects of GBS. In this regard, augmented Th17 cells and cytokines are known to be associated with EAN severity.^34^ M1 macrophages were also found to aggravate EAN via boosting Th17 response by secreting exosomes.^35^ In COVID‐19, augmented Th17 response has been observed during the cytokine storm.^36^ Lymphocytes isolated from patients with COVID‐19 were also found to produce more IL‐17.^37^ These findings together with our bioinformatic analyses support that the augmented Th17 cell differentiation and cytokine response in COVID‐19 may hasten GBS development.

A major limitation of the current study is that the identified gene networks mediating COVID‐19‐GBS interactions require further confirmation with experimental animal models. For instance, mouse models of COVID‐19 (eg by adeno‐associated virus–mediated expression of human ACE2 in the respiratory tract followed by experimental SARS‐CoV‐2 infection) and GBS (eg EAN induction by sensitization with peripheral nerve homogenate or by immunization with myelin proteins) could be combined to study the immunopathogenesis of COVID‐19–associated GBS.^38^, ^39^ In experimental settings, treatments (eg atorvastatin, retinoic acid receptor–related orphan nuclear receptor γt inhibitors, Bifidobacterium, Class I PI3K inhibitor ZSTK474, 2‐deoxy‐D‐glucose) that decrease Th17 cell number or cytokines have been shown to ameliorate EAN.^40^, ^41^, ^42^, ^43^, ^44^ It would be interesting to investigate whether some of these agents (eg atorvastatin or Bifidobacterium) could be used for preventing or treating COVID‐19–associated GBS.

## 5 CONCLUSION

We first defined the genes involved in GBS and COVID‐19, followed by delineating the protein‐protein interaction networks. Augmented Th17 cell differentiation and cytokine response was identified as an important process connecting COVID‐19 to GBS. PBMC transcriptome analysis also suggested the pivotal involvement of Th17 signalling pathway. In conclusion, our data suggested aberrant Th17 cell differentiation as a central mechanism by which COVID‐19 heightened the risk of GBS.

## CONFLICT OF INTEREST

The authors declare that they have no conflict of interest.

## AUTHORS CONTRIBUTIONS

Zheng Li, Xingye Li, Cheng Huang, Shugang Li and Jianxiong Shen conceptualized the study and analysed the data. Lin Zhang, Ziheng Huang and William Ka Kei Wu performed the bioinformatic analysis. Zheng Li, Sunny H. Wong, Matthew TV Chan and William Ka Kei Wu drafted and revised the manuscript. All authors critically reviewed the draft and approved the final manuscript.

## Data Availability

Research data will be shared upon request.
